# Celebrating Dr. Katsuhiko Ariga’s 60th birthday: from Nanotechnology to Nanoarchitectonics

**DOI:** 10.1080/14686996.2025.2532290

**Published:** 2025-08-01

**Authors:** Masanobu Naito, Ajayan Vinu, Yusuke Yamauchi

**Affiliations:** a Research Center for Macromolecules and Biomaterials, National Institute for Materials Science, Ibaraki, Japan; b Global Innovative Centre for Advanced Nanomaterials, College of Engineering, Science and Environment, School of Engineering, The University of Newcastle, Callaghan, NSW, Australia; c Department of Materials Process Engineering, Graduate School of Engineering, Nagoya University, Nagoya, Aichi, Japan; d Australian Institute for Bioengineering and Nanotechnology (AIBN) & School of Chemical Engineering, The University of Queensland (UQ), Queensland, Australia

## 1. From self-assembly to Nanoarchitectonics

This special issue of Science and Technology of Advanced Materials (STAM) commemorates the 60th birthday of Professor Katsuhiko Ariga, an internationally renowned scientist whose groundbreaking contributions in supramolecular chemistry, interfacial science, and materials design have profoundly shaped the field of advanced materials. His academic journey spans from foundational studies in molecular self-assembly – including pioneering work on synthetic bilayer membranes – to the development and global dissemination of the now widely embraced concept of nanoarchitectonics.

Prof. Ariga earned his M.S. (1987) and Ph.D. (1990) from the Tokyo Institute of Technology. He began his academic career as an Assistant Professor there (1987–1992) and conducted postdoctoral research at the University of Texas at Austin (1990–1992). He then served as Project Leader for the JST Supermolecules Project (1992–1997), followed by positions as Associate Professor at NAIST (1998–2001) and Group Leader in the JST Aida Nanospace Project (2001–2003). Since 2004, he has been a Group Leader at the National Institute for Materials Science (NIMS), and in 2007 he became a Principal Investigator at MANA. In 2017, he was appointed as Professor at the University of Tokyo.

Prof. Ariga’s early achievements laid the foundation for supramolecular materials science. His research into synthetic bilayer membranes and self-assembly phenomena provided fundamental insights into the organization and function of nanoscale systems. These principles were later extended through his innovations in Layer-by-Layer (LbL) assembly, which evolved into a practical and versatile platform for nanofilm fabrication, finding applications in sensors, catalysis, and biointerfaces. Capitalizing on these foundations, Prof. Ariga introduced and advanced the paradigm of nanoarchitectonics – a methodology for constructing novel materials by orchestrating nanoscopic components (atoms, molecules, and nanoparticles) through controlled self-organization and interface design.

In contrast to traditional top-down microfabrication techniques, nanoarchitectonics embraces the complex, stochastic, and interactive nature of nanoscale systems. Its four core principles include: (1) Unreliability-tolerant reliability – harnessing thermal and statistical fluctuations to realize robust nanoscale functions; (2) From nano-functionality to nanosystem-functionality – creating emergent properties through synergistic interactions among diverse nanocomponents; (3) Quantity changes quality – exploiting scale-dependent complexity to generate functionalities absent in isolated constituents; and (4) Truth can be described with plain words – promoting bold, intuitive frameworks alongside rigorous atomistic principles.

Within this framework, Prof. Ariga developed a broad and impactful research portfolio encompassing molecular recognition, interfacial nanostructures, energy materials, biomedical applications, and soft electronics. Today, nanoarchitectonics stands as a globally recognized and unifying concept in materials science, reshaping our understanding and design of complex functional systems.

## 2. Editorial leadership in STAM

Prof. Ariga has also been playing a pivotal role in STAM’s growth since his appointment as an Associate Editor in 2007. His influence extends well beyond routine manuscript handling: he has actively steered the journal’s editorial vision by spearheading impactful review articles and by organizing thematic Focus Issues that highlight pioneering ideas in emerging areas of materials science. Among the Focus Issues he has led are:

‘Future Leaders in Nanoarchitectonics’ (2015) – showcasing rising stars and new directions in nanoarchitectonics research.

‘Advancements of Functional Materials with Nanoarchitectonics as Post-Nanotechnology Concept in Materials Science’ (2022–2023) – exploring how nanoarchitectonics extends and transforms functional materials design in the post-nanotechnology era.

‘Nanoarchitectonics Reloaded: Method for Everything in Materials Science’ (2023–2025) – reinvigorating the discussion on nanoarchitectonics as a universal methodology across diverse materials domains.

These efforts have positioned STAM as a leading platform for conceptual and interdisciplinary innovation in materials science. In recognition of his service, Prof. Ariga received the STAM Best Contribution Award in 2017 for his exceptional editorial leadership and dedication.

## 3. Contributions as author in STAM

In parallel with shaping the journal’s direction, Prof. Ariga has been a prolific contributor to STAM as an author. To date he published 17 scholarly works in STAM [1–17], comprising 8 review papers and 9 original research articles, which collectively have been cited over 1700 times. These contributions span nearly two decades (from Volume 7 in 2006 to Volume 25 in 2024) and encompass a wide range of topics – from porous materials and self-assembly to biomaterials and nanoarchitectonic strategies – reflecting his evolving research interests and the breadth of his expertise.

Notably, one of his early reviews, ‘Challenges and breakthroughs in recent research on self-assembly’ (2008) became a landmark in the field, being ranked the #1 Hot Paper in Materials Science in 2010 and remaining one of the most highly cited papers in STAM’s history [2]. Many of his STAM publications have similarly made outsized impact.

Several of Prof. Ariga’s STAM papers have been honored with Best Paper Awards. For example, his seminal self-assembly review (published 2008) was recognized with STAM’s Best Paper Award in 2010 [2]. A collaborative research article reporting the formation of metal clusters in halloysite clay nanotubes (published 2017) received the 2018 Best Paper Award [7]. More recently, his 2019 review ‘Self-assembly as a key player for materials nanoarchitectonics’ earned the 2021 Best Paper Award [8]. These accolades underscore the influence of his work: the 2008 self-assembly review alone, for instance, has been cited on the order of 700 times [2], attesting to its fundamental importance. Each of these award-winning papers not only expanded the frontiers of knowledge but also exemplified the journal’s highest standards of excellence.

## 4. Mentorship and legacy

Prof. Ariga’s dedication to collaboration and mentorship has fostered an enduring network of partnerships across disciplines and around the world. His openness and visionary ideas have catalyzed the birth of numerous interdisciplinary endeavors. This special issue itself embodies the respect and appreciation of his global community of colleagues and protégés – many of whom have contributed articles with heartfelt enthusiasm to celebrate his 60th birthday.

Notably, the issue features works from researchers who trained under his mentorship as graduate students or postdoctoral fellows or research scientists. Having internalized his scientific philosophy – characterized by conceptual clarity, molecular-level intuition, and integrative thinking – they now carry his influence into diverse research arenas. Their participation in this issue stands as a testament not only to his professional leadership but also to his personal impact as an inspiring mentor and role model.

Prof. Katsuhiko Ariga’s legacy is defined by its originality, breadth, and lasting impact. From early breakthroughs in self-assembly to the establishment of nanoarchitectonics as a transformative paradigm, his scientific journey has fundamentally reshaped how we envision, design, and utilize functional materials. It is both an honor and a privilege for us to dedicate this special issue of STAM to him on the occasion of his 60^th^ birthday. We offer our deep admiration and celebratory best wishes to Prof. Ariga, whose visionary contributions will continue to inspire future generations of scientists and illuminate the path toward new frontiers in materials innovation.
